# Cytotoxic Components of *Pereskia bleo* (Kunth) DC. (Cactaceae) Leaves

**DOI:** 10.3390/molecules14051713

**Published:** 2009-05-06

**Authors:** Sri Nurestri Abdul Malek, Sim Kae Shin, Norhanom Abdul Wahab, Hashim Yaacob

**Affiliations:** 1 Institute of Biological Sciences, Faculty of Science, University of Malaya, 50603 Kuala Lumpur, Malaysia; 2 Institute of Postgraduate Studies, University of Malaya, 50603 Kuala Lumpur, Malaysia; 3 International University College of Nursing, B-27-6, Block B, Jaya One, No 72A Jalan Universiti, 46000 Petaling Jaya, Selangor, Malaysia

**Keywords:** *Pereskia bleo*, Cactaceae, cytotoxic activity, cell lines

## Abstract

Dihydroactinidiolide (**1**) and a mixture of sterols [campesterol (**2**), stigmasterol (**3**) and β-sitosterol (**4**)], together with the previously isolated individual compounds β-sitosterol (**4**), 2,4-di-tert-butylphenol (**5**), α-tocopherol (**6**), phytol (**7**) were isolated from the active ethyl acetate fraction of *Pereskia bleo* (Kunth) DC. (Cactaceae) leaves. Cytotoxic activities of the above mentioned compounds against five human carcinoma cell lines, namely the human nasopharyngeal epidermoid carcinoma cell line (KB), human cervical carcinoma cell line (CasKi), human colon carcinoma cell line (HCT 116), human hormone-dependent breast carcinoma cell line (MCF7) and human lung carcinoma cell line (A549); and non-cancer human fibroblast cell line (MRC-5) were investigated. Compound **5** possessed very remarkable cytotoxic activity against KB cells, with an IC_50 _value of 0.81µg/mL. This is the first report on the cytotoxic activities of the compounds isolated from *Pereskia bleo*.

## Introduction

The leaves of *Pereskia bleo* (Kunth) DC. (Cactaceae) are used traditionally in Malaysia for the treatment of cancer, high blood pressure, diabetes and diseases associated with rheumatism and inflammation. They are also used as remedy for the relief of gastric pain, ulcers and for revitalizing the body [1]. The leaves are generally consumed by the locals either raw or taken as a concoction brewed from fresh leaves. 

Chemical investigations on *Pereskia bleo* are rare in comparison to other *Pereskia* species, as there were only three phytochemical and biological studies reported for this plant. The earliest phytochemical study was by Doetsch *et al.* [4], who reported the isolation of four alkaloids, namely 3,4-dimethoxy-β-phenethylamine, mescaline, 3-methoxytyramine and tyramine. An investigation by Tan *et al.* [2] reported that the methanol extract of *Pereskia bleo* possessed cytotoxic effects against T-47D cells and the cell death was found to be apoptotic in nature, mainly *via* the activation of the caspase-3 and c-myc pathways. A more recent investigation by Er *et al*. [3] indicated the anti-proliferative and mutagenic activities of aqueous and methanol extracts of *Pereskia bleo* leaves against mouse mammary cancer cells (4T1) or normal mouse fibroblast cells (NIH/3T3). In our previous cytotoxicity screenings on *Pereskia bleo* [5], the EtOAc fraction possessed notably high cytotoxic effects against selected human carcinoma cell lines, but exerted no damage to a non-cancer human fibroblast cell line (MRC-5). The active EtOAc fraction was found to contain β-sitosterol (**4**), 2,4-di-tert-butylphenol (**5**), α-tocopherol (**6**) and phytol (**7**) [5]. As part of our ongoing research on *Pereskia bleo*, a pure compound and a mixture of sterols were also isolated from the leaves of *Pereskia bleo*.

In the present study, we report further progress in ongoing research on *Pereskia bleo*, which led to the isolation and identification of dihydroactinidiolide (**1**) and a mixture of sterols [campesterol (**2**), stigmasterol (**3**) and β-sitosterol (**4**)] and cytotoxic investigation on all isolated compounds against five human carcinoma cell lines, namely the human nasopharyngeal epidermoid carcinoma cell line (KB), human cervical carcinoma cell line (CasKi), human colon carcinoma cell line (HCT 116), hormone-dependent breast carcinoma cell line (MCF7) and human lung carcinoma cell line (A549) and non-cancer human fibroblast cell line (MRC-5).

## Results and Discussion

### Extraction and isolation of pure compounds and the sterol mixture

β-Sitosterol (**4**), 2,4-di-tert-butylphenol (**5**), α-tocopherol (**6**) and phytol (**7**) were obtained from *Pereskia bleo* as previously described by Sri Nurestri *et al.* [5]. On repeated chromatographic purification of the active EtOAc fraction, a red viscous oil and white colored needles were obtained and identified as dihydroactinidiolide and a mixture of sterols.

Dihydroactinidiolide (**1**), red viscous oil; EI-MS *m/z* (%): 180 [M]^ +^ (15), 137 (8), 111 (100), 109, 67. Compound **1 **was identified by comparison of its mass spectral data with NIST mass-spectral library [21] and other reported spectroscopic data [6,7,8]. 

**Figure 1:**
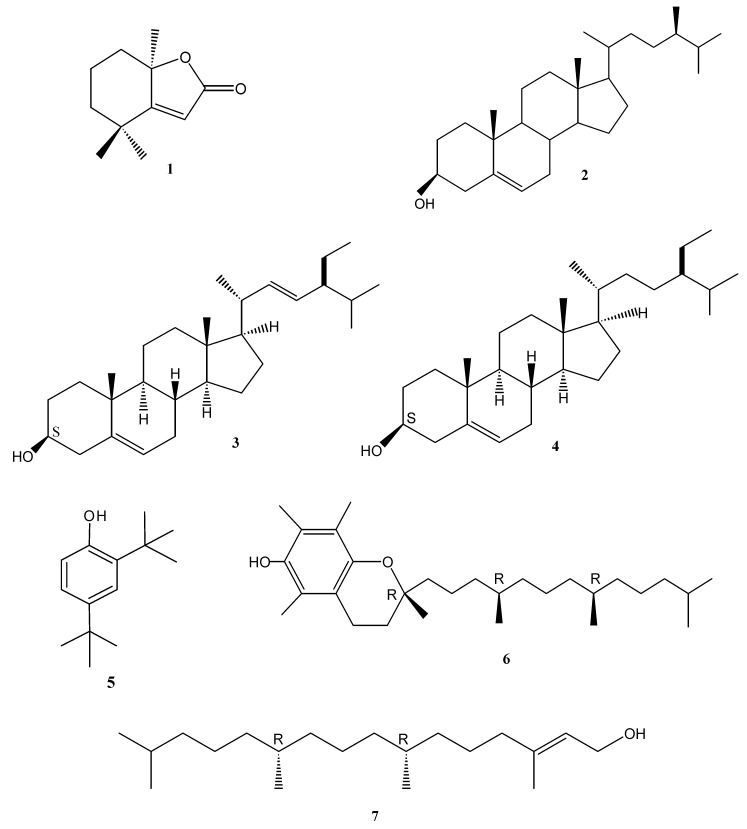
Structures of compounds **1-7**.

The mixture of sterols appeared as white colored needles that according to GC-MS analyses consisted of campesterol (**2**, 14.33%), stigmasterol (**3**, 4.95%) and β-sitosterol (**4**, 70.21%). Compound **2 **(campesterol); EI-MS *m/z* (%): 400 (42, [M^+^]), 382 (34), 367 (20), 315 (30), 289 (30), 55 (100). The mass spectral data was also in agreement with reported data [9]. Stigmasterol (**3**) was identified by GC-MS analysis and by comparison of its mass spectral data [EI-MS *m/z* (%): 412 (16, [M^+^]), 394 (4), 369 (2), 351 (6), 271 (16), 255 (22), 229 (5), 55 (100)] with reported data [9]. Compound **4 **(ß-sitosterol); EI-MS *m/z* (%): 414 (100, M^+^), 396 (57), 381 (43). ß-sitosterol (**4**) was identified by GC-MS analysis as well as comparison of its mass spectral data with reported data [10]. The structures of compounds **1**-**7** are illustrated in Figure 1.

### In vitro Neutral Red cytotoxicity assay

The *in vitro* cytotoxicity assay was carried out using a Neutral Red cytotoxicity assay as previously described by Borenfreund and Puerner [11] with some modifications; this test determines the accumulation of the Neutral Red dye in the lysosomes of viable and uninjured cells. 

The results of cytotoxicity screening of the components are summarized in Table 1. It is generally known that ethnomedical data substantially increases the chances of finding active plants relative to a random approach [2]. The consequence is that, once having found activity in the plant from the ethopharmacological observation (e.g. raw or concoction brewed from the plant leaves shows effect for cancer treatment), there is a desire to determine the chemical structures of the compounds that are responsible for the activity, as not all the compounds in the extracts have the same activity. 

However, the observed activity might be due to synergism between compounds present in the plant extract. The synergism among these compounds which contribute to the cytotoxic activity, is not only dependent on the concentration of the compounds, but also on the structure and interaction(s) between the compounds [27]. This can explain the differences in the cytotoxic effect between crude extracts and isolated compounds against the same cell lines, as shown in our earlier report [5]. For example, the cytotoxic effect of the crude methanol extract on the KB cell lines showed an IC_50_ of 6.5 µg/mL and such impressive activity was supported by some of the isolated compounds [dihydroactinidiolide (**1**), 2,4-di-tert-butylphenol (**5), **α-tocopherol (**6**) and phytol (**7**)]. In contrast, the cytotoxic effect of the crude methanol extract on the MCF7 cell line gave IC_50_ of 39.0 µg/mL (mild) whilst two isolated compounds 2,4-di-tert-butylphenol (**5)** and α-tocopherol (**6**), showed good inhibitory activities with IC_50_ values of 5.75 and 7.5 µg/mL, respectively.

**Table 1:** Cytotoxic activity (IC_50 _values) of compounds **1**-**5** and mixture of sterols against KB, CasKi, HCT 116, MCF7, A549 and MRC5 cell lines.

Compound	Cytotoxicity (IC_50_) in µg/mL (µM)					
	KB	MCF7	CasKi	HCT 116	A549	MRC-5
Dihydroactinidiolide (**1**)	6.7	30	40	5	97	91.3
	(37.22)	(166.67)	(222.22)	(27.78)	(538.89)	(507.22)
β -sitosterol (**4**)	>100	72	62	>100	78	>100
	(>241.55)	(173.91)	(149.76)	(>241.55)	(188.41)	(>241.55)
2,4-di tert butylphenol (**5**)	0.81	5.75	4.5	29	6	20
	(3.93)	(27.91)	(21.84)	(140.78)	(29.13)	(97.09)
α-tocopherol (**6**)	8	7.5	6	31	6	30.5
	(18.6)	(17.44)	(13.95)	(72.09)	(13.95)	(70.93)
Phytol (**7**)	7.1	34	18	100	31	74.3
	(23.99)	(114.86)	(60.81)	(337.84)	(104.73)	(251.01)
Mixture	>100	>100	>100	>100	>100	>100
Doxorubicin^a^	1.3x10^-2^	7.6x10^-2^	6.0x10^-3^	3.6x10^-1^	2.2x10^-1^	5.5x10^-1^
	(0.023)	(0.139)	(0.011)	(0.663)	(0.401)	(1.01)

^a ^Doxorubicin was used as the reference compound.

2,4-Di-tert-butylphenol (**5)** displayed very remarkable cytotoxic activity against KB cells with an IC_50 _value of 0.81 µg/mL and strong cytotoxicity against MCF7 (IC_50_ 5.75 µg/mL), A549 (IC_50_ 6 µg/mL) and CasKi cells (IC_50 _4.5 µg/mL). This *in vitro* data of 2,4-di-tert-butylphenol (**5**) support the findings that phenolic antioxidants exert cytoctoxic activity against cancer cells [14,15]. 2,4-Di-tert-butylphenol (**5**) is an antioxidant widely used in the plastics industries, and its presence in plants cannot readily be explained biogenetically. It is more probable that the plant accumulated this compound from the soil it grew in, that might have contained the compound. In our experience, this compound has also been detected in other plants like *Termitomyces heimi*, *Pleurotus sajor-caju* and *Hericium erinaceus* collected from different locations to where the *Pereskia bleo* leaves were obtained (unpublished data from our group of researchers working on *Termitomyces heimi*, *Pleurotus sajor-caju* and *Hericium erinaceus*). The observation of 2,4-di-tert-butylphenol (**5**) in our study is not an isolated case, as it has also been reported to exist in natural sources by other researchers [29,30,31]. To support our finding that 2,4-di-tert-butylphenol (**5**) is not an artifact, an extraction on *Pereskia bleo* was repeated using redistilled methanol and ethyl acetate. GC-MS analysis on the ethyl acetate extract still showed the presence of 2,4-di-tert-butylphenol (**5**) representing the major component of the total ethyl acetate extract. This shows that 2,4-di-tert-butylphenol (**5**) is present in the extract itself and not a solvent artifact. 

Other constituents in the plant also contribute to its cytotoxic activity as shown by α-tocopherol (**6**), phytol (**7**) and dihydroactinidiolide (**1**). In the present study, α-tocopherol (**6**), which is a dietary antioxidant, displayed pronounced cytotoxicity against CasKi (IC_50_ 6 µg/mL) and A549 (IC_50_ 6 µg/mL). The result obtained here is consistent with other reports [37,38,39,40,47,48,49,50] on cytotoxic activities in other cell lines. Lesser number of investigations described an opposite effect [44,45,46]. There was no report on the cell lines that were used in this study. According to Table 1, phytol (**7**) demonstrated strong activity against KB cells (IC_50_ 7.1 µg/mL). The cytotoxicity data showed in this report thus supports our hypothesis in our previous report [5] that phytol might be responsible for the remarkable cytotoxic effect of the EtOAc fraction against the KB cancer cell lines. In this study, dihydroactinidiolide (**1**) demonstrated strong cytotoxic effect against HCT116 with IC_50_ 5.0 µg/mL. Dihydroactinidiolide (**1**) is structurally similar to the C11-terpene lactones that arise from the biological or oxidative degradation of carotenoids and has been isolated from various plants and insect sources. It has also been identified as the flavor molecule in tea and tobacco [6,7,8].

Sterols are important constituents of all eukaryotes and play a vital role in plant cell membranes. In addition to their cholesterol lowering effect, plant sterols may possess anti-atherosclerosis [32,33], antibacterial [36], anti-inflammation [34] and anti-oxidation activities [35]. In the present study, β-sitosterol (**4**) and the mixture of sterols [campesterol (**2**), stigmasterol (**3**) and β-sitosterol (**4**)] did not display cytotoxic effects against the tested cell lines. The results obtained here were in agreement with published data [16,17,18,19,20]. There have been reports that plant sterols are able to stimulate estrogen dependent cancer cells *in vitro* (e.g. Ju *et al*. [42]). The MCF7 cell line used in this study was purchased from ATCC. It was reported that MCF7 cells from ATCC were unaffected by estrogen or antiestrogen [43]. Thus, the result showed that the sterols do inhibit the growth of MCF7 cells. 

Doxorubicin which is clinically used for the treatment of a great variety of cancer disease [24,25,26] was used as the positive control in present study. Based on the result, it can be concluded that doxorubicin is not only cytotoxic against all the human cancer cell lines tested, but also the non-cancer human cell line. This result supports the statement that doxorubicin is a potent cytostatic drug which is applied for the treatment of cancer diseases but the routine use of this drug could bring major adverse effect [24]. Although the cytotoxicity of the isolated compounds and mixture of *Pereskia bleo* are not as effective as doxorubicin, they however have low toxicity against normal MRC5 cell line in comparison to doxorubicin. The use of the isolated compounds as single anticancer agents would not merit consideration. However, their use in combination with cytotoxic therapeutic drugs might reduce the adverse effects of some of these drugs. Support for this suggestion is provided by Amir *et al.* [41], who reported that in addition to having potent antitumor properties as single agents, natural products have also demonstrated potential synergy with established cytotoxic therapeutic drugs in pre-clinical studies. At this stage, it is not possible to justify the use of isolated compounds in comparison to doxorubicin in the treatment of cancer. A more comprehensive investigation is required. 

## Experimental

### General

GCMS analysis was performed using a Agilent Technologies 6980N gas chromatography equipped with a 5979 Mass Selective Detector (70 eV direct inlet); a HP-5ms (5% phenylmethylsiloxane) capillary column (30.0 m x 250 µm x 0.25 µm) initially set at 60°C for 10 minutes, then programmed to 230°C at 3°C min^-1^and held for 1 min at 230°C using helium as the carrier gas at a flow rate of 1 mL min^-1^. The total ion chromatogram obtained was auto integrated by ChemStation and the components were identified by comparison with an accompanying mass spectral database [21]. Thin layer chromatography (TLC) analyses were carried out using precoated TLC plates 60 F_254_ (20.25 mm thickness) purchased from Merck and were visualized in UV light (254 and/or 343 nm) and/or iodine vapour.

### Plant sample collection and identification

The fresh leaves of *Pereskia bleo* were collected from Petaling Jaya, Selangor, Malaysia in September 2006. They were identified by Professor Dr. Halijah Ibrahim of Institute of Biological Sciences, Faculty of Science, University of Malaya, Malaysia and a voucher specimen (SN01-06) was deposited at the herbarium of the Institute of Biological Sciences, Faculty of Science, University of Malaya, Kuala Lumpur, Malaysia.

### Extraction and isolation of pure compound and mixture

β-Sitosterol (**4**), 2,4-Di-tert-butylphenol (**5**), α-tocopherol (**6**) and phytol (**7**) were isolated from *Pereskia bleo* as previously described by Sri Nurestri *et al.* [5]. Compound **1** and mixture of sterols were obtained according to the following procedure. Dried, ground leaves (1,050.56 g) of *Pereskia bleo* were extracted with MeOH (3x 1.5 L). The MeOH-containing extract obtained was initially treated with charcoal, then filtered over Celite^®^ and the filtrate was evaporated under reduced pressure to give a crude MeOH extract (99.44 g). Treatment with charcoal was necessary to remove the high amounts of chlorophyll present in the extract, which interfered with chromatographic separation efforts. The crude MeOH extract was then further partitioned between EtOAc and H_2_O in a separating funnel. The EtOAc-soluble layer was concentrated *in vacuo* giving an 18.34 g EtOAc fraction, which was subjected to flash silica gel column chromatography (Si-gel CC) eluting with CHCl_3_ (10 L), and then with CHCl_3_-MeOH [9:1 (9 L)] and finally MeOH (7.6 L). The CHCl_3_ fraction was concentrated to give a dark brown residue (3.47 g). The brown residue was subjected to a Si-gel CC initially eluting with a gradient of hexane followed by hexane enriched with increasing percentages of CH_2_Cl_2_, monitoring with TLC. The volume of each fraction was 25 mL. The mixture of sterols (20.5 mg) was obtained from the fraction upon elution with CH_2_Cl_2_-hexane (3.5: 6.5). Further elution with CH_2_Cl_2_ yielded a mixture (206.7 mg) containing **1**. Purification of **1 **was obtained through preparative-TLC using CHCl_3_ as the developing solvent to yield pure compound **1 **(5.4 mg). 

### Cell lines and culture medium

Human nasopharyngeal epidermoid carcinoma cell line (KB), human cervical carcinoma cell line (CasKi), human colon carcinoma cell line (HCT 116), human hormone-dependent breast carcinoma cell line (MCF7), human lung carcinoma cell line (A549) and non-cancer human fibroblast cell line (MRC-5) were purchased from the American Tissue Culture Collection (ATCC, USA). KB cells were maintained in Medium 199 (Sigma), CasKi, A549 and MCF7 cells in RPMI 1640 medium (Sigma), HCT 116 in McCOY’S 5A Medium (Sigma) and MRC-5 cells in EMEM (Eagle Minimum Essential Medium) (Sigma), supplemented with 10% foetal bovine serum (FBS, PAA Lab, Austria), 100 µg/mL penicillin or streptomycin (PAA Lab, Austria) and 50 µg/mL of fungizone (PAA Lab, Austria). The cells were cultured in a 5% CO_2_ incubator (Shel Lab water-jacketed) kept at 37°C in a humidified atmosphere. 

### In vitro Neutral Red cytotoxicity assay

The Neutral Red cytotoxicity assay is based on the initial protocol described by Borenfreund and Puerner [11] with some modifications. Briefly, the cells (1x10^4^/well) were seeded in 96-well microtiter plates (Nunc) and allowed to grow for 24 hours before treatment. After 24 hours of incubation, the cells were treated with six different concentrations (0.1-100 µg/mL) of test compounds, in three replicates. The plates were further incubated for 72 h at 37°C in a 5% CO_2_ incubator. A stock solution was initially obtained by dissolving the test compounds in DMSO. Further dilution to different tested concentrations were then carried out ensuring that the final concentration of DMSO in the test and control wells was not in excess of 1% (v/v). No effect due to the DMSO was observed. Doxorubicin was used as the positive control. The well containing untreated cells was the negative control. At the end of the incubation period, the media were replaced with medium containing 50 µg/mL of Neutral Red. The plates were incubated for another 3 hours to allow for uptake of the vital dye into the lysosomes of viable and injured cells. After the incubation period, the media were removed and cells were washed with the neutral red washing solution. The dye was eluted from the cells by adding 200 µL of Neutral Red resorb solution and incubated for 30 minutes at room temperature with rapid agitation on a microtiter plate shaker. Dye absorbance was measured at 540 nm using a spectrophotometer ELISA plate reader. The average data from triplicates were expressed in terms of killing percentage relative to negative control. The percentage of inhibition (%) of each of the test samples was calculated according to the following formula:

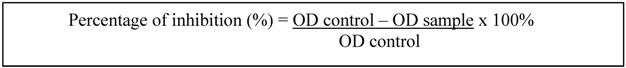

where OD control: Optical Density of negative control; OD sample: Optical Density of sample

Cytotoxicity of each sample is expressed as IC_50_ value. The IC_50 _value is the concentration of test compounds that cause 50 % inhibition or cell death, averaged from the three experiments, and was obtained by plotting the percentage inhibition versus concentration of test compounds. According to US NCI plant screening program, a plant extract is generally considered to have active cytotoxic effect if the IC_50_ value, following incubation between 48 to 72 hours, is 20 μg/mL or less, while it is 4 μg/mL or less for pure compounds [12,13,22,23]. However, we recognized that whether an IC_50_ value corresponds to a significant or non-significant cytotoxicity depends on the sensitivity of the cell line. 

## Conclusions

In conclusion, and depending on the cell lines used, the cytotoxic activities observed for *Pereskia bleo* [5] are ascribable to the presence of the active compounds **1**, **5**, **6** and **7**. Although the cytotoxicity of these compounds and mixture are not as effective as doxorubicin, in comparison to the latter they have low toxicity against normal MRC5 cell line. The cytotoxicity assay used in the present study could only provide important preliminary data to help select plant extracts or isolated compounds with potential antineoplastic properties for future work. A detailed investigation on the mechanism of cell death would provide more convincing evidence. An investigation into this phenomenon is now underway and will be reported in due course. The resulting information will certainly contribute to a better understanding of the anti-carcinogenic activity of the natural constituents in *Pereskia bleo*. 

*Pereskia bleo* has been traditionally used for the treatment of cancer and the findings of the current study thus provide scientific validation on the use of the leaves of *Pereskia bleo*. In view of the increasing popular consumption of medicinal plants as alternative therapy, it is therefore necessary to conduct serious research to support the therapeutic claims and also to ensure that the plants are indeed safe for human consumption.
